# Preparation of silica coatings with continuously adjustable refractive indices and wettability properties *via* sol–gel method†

**DOI:** 10.1039/c7ra12817g

**Published:** 2018-02-07

**Authors:** Bibo Xia, Lianghong Yan, Yuanyang Li, Shuming Zhang, Meiying He, Hao Li, Hongwei Yan, Bo Jiang

**Affiliations:** Key Laboratory of Green Chemistry & Technology, College of Chemistry, Sichuan University Chengdu 610064 P. R. China jiangbo@china.com; Research Center of Laser Fusion, China Academy of Engineering Physical Mianyang 621900 P. R. China hwyan@163.com

## Abstract

Silica coatings with continuously adjustable refractive indices and wettability properties were prepared through a sol–gel base-catalyzed process. Adjustment of the molar ratio of water (H_2_O) to tetraethylorthosilicate (TEOS) was utilized to change the hydrolysis degree of the precursors, and hence change the morphology of the silica particles. With the increase in the H_2_O/TEOS molar ratio, the morphology of the silica particles changed from a linear net-work structure to a bead-like structure and then to a granular particle structure. A particle growth mechanism was proposed and verified by characterization. As the H_2_O/TEOS molar ratio increased from 0.3 to 21.0, the refractive indices of the silica coatings increased from 1.132 to 1.328. Meanwhile, a varied H_2_O/TEOS molar ratio also modulated the surface wettability of the silica coatings. The static water angle of the silica coatings decreased from 145° to 6° by increasing the H_2_O/TEOS molar ratio from 0.3 to 21.0. Different hydrophilic and hydrophobic coatings could be obtained by simply controlling the H_2_O/TEOS molar ratio. Silica coatings with different refractive indices and hydrophobic (or hydrophilic) properties were obtained at different H_2_O/TEOS molar ratios.

## Abstract 2

Silica coatings with continuously adjustable refractive indices and wettability properties were prepared through a sol–gel base-catalyzed process.

## Graphical abstract

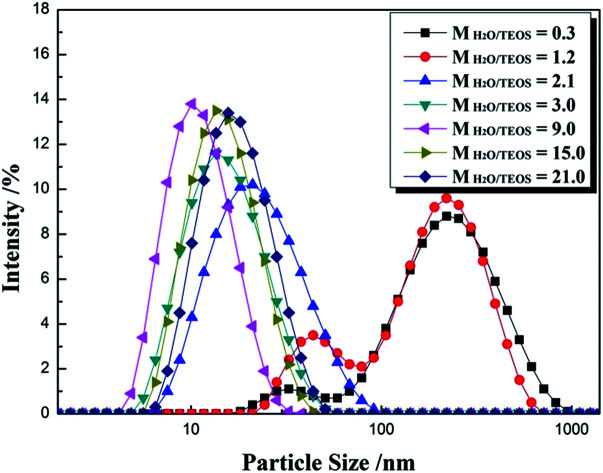

## Introduction

Antireflective (AR) coatings have been widely used in optical devices and energy-related applications.^1–4^ The principle of AR is the destructive interference between light reflected from the coating-substrate and the air-coating interfaces. The reflective index of coating (*n*_c_) and actual thickness of coating (*d*_c_) are decisive factor for obtaining an ideal homogeneous AR coating which achieves effectively 100% transmittance at a specific wavelength, that is, *n*_c_ is equal to (*n*_a_*n*_s_)^0.5^, where *n*_a_ and *n*_s_ are the refractive indices of the air and the substrate, respectively; and *d*_c_ should be *λ*/4*n*_c_, where *λ* is the wavelength of incident light.^5,6^ A typical glass has an index of refraction between 1.45 and 1.65 in the visible spectral region, which implies that the index of refraction of the antireflective interference film must be between 1.20 and 1.25. Furthermore, a coating with wide-ranged refractive indices could be a good choice for multilayer wide-range AR coatings.^7,8^ There are several approaches to introduce antireflection,^9–15^ and among them, depositing a coating with determined refractive index on a specific substrate is a widely used method, therefore it is of great significance to fabricate coatings with controllable refractive indices in a relatively wide range.

Among the industrial-viable methods to produce these coatings, sol–gel chemistry is one of the most interesting method because of its low process temperature, low cost, the high purity of the resulting materials, easy to combine with liquid deposition techniques that permit an accurate control of the coating thickness and the adaptability towards substrates with various shapes and sizes.^16,17^ Acid-catalyzed sol–gel method has been carried out to prepare silica coatings with varied refractive index a lot, but base-catalyzed has not drawn researcher's attention. Zhang and co-workers reported hexamethyldisilazane-modified base-catalyzed silica coating with refractive indices ranging from 1.23 to 1.13.^7^ Ye obtained silica coating with refractive indices from 1.42 to 1.25 by mixing acid-catalyzed silica sol with base-catalyzed silica sol.^18^ However, they are both multi-steps process, which boosts the time and cost of coatings preparation in industrial applications. Zhang Yulu demonstrated that by using the co-condensation of tetraethylorthosilicate (TEOS) and methyltriethoxysilane (MTEOS), the refractive indices of prepared silica coatings are controllable from 1.21 to 1.10.^19^ The above mentioned methods are all derived from Stöber process base-catalyzed silica sol,^20^ so the refractive indices of prepared coatings are limited by that of Stöber process. Thus, new fabrication methods to prepare silica coating with refractive index value varying in a wide range are of great interest.

Wettability is also an important property of the coating because it can have great impact on the coating durability during its use. Hydrophobic or hydrophilic property draw much attention about coating's wetting behavior.^21–25^ As an essential aspect of surface chemistry, the wettability control of the coating surface shows enormous value in both fundamental research and practical applications.^26–28^ Two crucial factors have been demonstrated to explain the surface wetting behavior.^29,30^ One is the surface roughness represented by various periodically or randomly distributed micro/nanostructures, and the other is the surface chemical composition. According to this basic principle various hydrophobic and hydrophilic surfaces have been successfully prepared. Smart coatings with switchable wettability between hydrophobicity and hydrophilicity could satisfy the need of industrial application and laboratory investigation.^31,32^ AR coatings integrating with hydrophobic or hydrophilic property have fascinated a lot of researches.^33,34^ It is meaningful to fabricate AR coating whose surface wetting behavior is controllable.

In this work, silica coatings with wide-ranged controllable refractive indices and wettability properties were prepared by a simple method. The influence of the different H_2_O/TEOS molar ratio to base-catalyzed silica coatings was investigated systematically. As the molar ratio of H_2_O/TEOS varied, the morphology of silica particle showed linear net-work structure, bead-like structure and granular particle structure. The corresponding mechanism of particle growth were proposed and discussed.

## Experimental section

### Materials

Tetraethylorthoxylsilicane (TEOS, Si(OC_2_H_5_)_4_, 98%) was purchased from Acros Organics Company. Ethyl alcohol (EtOH, C_2_H_5_OH, 99.7%) and aqueous ammonia (NH_3_·H_2_O, 13.4 mol L^−1^) were purchased from Kelong Chemical Reagents Factory (Chengdu City, Sichuan Province, China). Ethyl alcohol was distilled twice before use. The water was deionized. All chemicals were used without further purification.

### Preparation of sols

First, TEOS was added into a sealable glass container precisely. Second, EtOH and H_2_O were added in the glass container and then immediately stirred for 10 min. Finally, NH_3_·H_2_O were added in the glass container. The final molar ratio of TEOS : EtOH : NH_3_ was 1 : 48 : 0.09, and sols with H_2_O/TEOS molar ratio (*M*_H_2_O/TEOS_) varying between 0.3–21.0 were prepared. The resultant sols were stirred for 2 hours at 30 °C and then aged in sealed glass containers at room temperature for various periods of time before deposition.

### Preparation of coatings

Fused silica substrates (refractive index of 1.46) were cleaned with ethanol in an ultrasonic bath, rinsed with deionized water and then wiped carefully before dip-coating. The silica sols were deposited on the well-cleaned fused silica substrates by dip-coating at varied withdrawal rate. The silica coatings were heat treated at 160 °C for 2 hours under ambient atmosphere.

### Characterization

To determine particle size and distribution, the silica sols were analyzed by dynamic light scattering (DLS, Malvern Nano-ZS) at 25 °C when aging time was 14 days. The transmission spectra were recorded using a UV-vis spectrophotometer (Mapada, UV-6300PC), with wavelength ranging from 400–1000 nm. Before transmission spectra measurement, the film thicknesses of AR coatings were optimized by varying the withdrawal rate and sol concentration. The structures of the silica colloids were investigated with a transmission electron microscope (JEOL, JEM-100CX) operated at 200 kV. The samples were prepared on a holey carbon coated copper grid by placing a drop of the colloidal suspension used for coatings. Scanning electron microscopy (SEM, JSM-5900LV) was used to observe the surface profile structures of coatings at low vacuum condition. Surface topography of the coatings was studied with atomic force microscopy (AFM, SEIKO SPA-400). The surface root-mean-square (RMS) roughness values were obtained from the analysis of atomic force microscopy (AFM) images. The refractive indices and thicknesses of coatings were determined using an ellipsometer (HORIBA UVISEL™) at incident angles of 70° at a wavelength of 632 nm. Static water contact angles of the coatings were performed using a contact angle meter (Krüss DSA100 Germany). FT-IR spectra of several xerogels were measured with a spectrometer Tensor 27 (Bruker, Germany) using KBr method in transmission mode.

## Results and discussion

### Particle size and coating characteristics

**Fig. 1:**
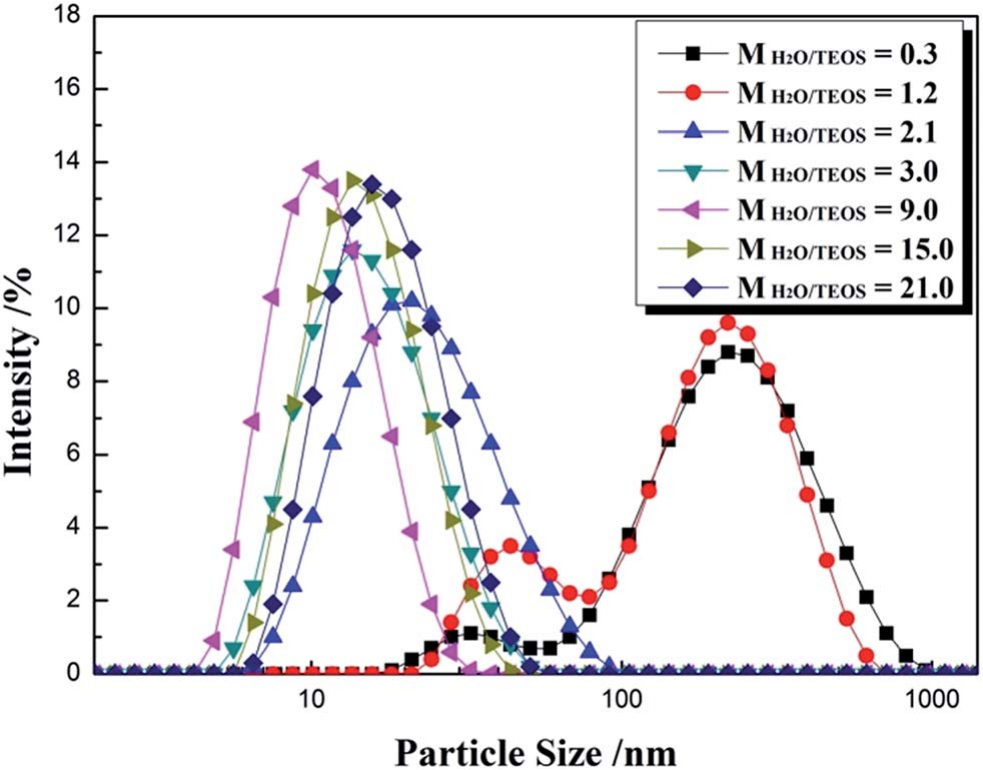
Particle size intensity of *M*_H_2_O/TEOS_ = 0.3, 1.2, 2.1, 3.0, 9.0, 15.0 and 21.0 silica sols.

For an AR coating prepared from sol–gel process, it is necessary to monitor the particle growth, which can be performed by the change of particle size and viscosity. For a given coating material, the values of refractive index are determined by the sol particle structure and the thickness is subject to the sol coating characteristics. Particle size and its distribution were investigated by DLS and are shown in Fig. 1. The intensity–size distribution exhibited an obvious bimodal pattern when the H_2_O/TEOS molar ratio value were 0.3 and 1.2, unimodal pattern were observed when the H_2_O/TEOS molar ratio value were higher than 2.1. While the H_2_O/TEOS molar ratio were 0.3, 1.2, 2.1, 3.0, 9.0, 15.0 and 21.0, their PdI (polydispersity index) values were 0.472, 0.418, 0.237, 0.226, 0.201, 0.183 and 0.155 respectively. So the sol particle with H_2_O/TEOS molar ratio of 0.3 and 1.2 were considered as polydispersed, the other sol particle were monodispersed.

Meanwhile, we tested the particle size of varied H_2_O/TEOS molar ratio silica sol as a function of aging time, as shown in Table 1. For the H_2_O/TEOS molar ratio value at 21.0, 15.0 and 9.0, the particle size of sol almost remained almost unchanged with aging time, the particle growth reached its ultimate stage within 4 days. As the H_2_O/TEOS molar ratio decreased, the particle size of silica sol became constant within longer aging time. When the H_2_O/TEOS molar ratio was 1.2 and 0.3, the particle size increased continuously within 14 days aging time. With the decrease of H_2_O/TEOS molar ratio, it needed longer aging time to reach the ultimate stage.

**Table 1:** Change in particle size with aging time at varied H_2_O/TEOS molar ratio

Aging time	The particle size (*d*/nm) of varied H_2_O/TEOS molar ratio						
	21	15	9	3	2.1	1.2	0.3
2 days	23.0	16.3	9.7	—	—	—	—
4 days	23.1	16.5	10.2	10.3	9.4	—	—
6 days	23.2	16.9	10.4	13.6	14.2	14.8	35.4
8 days	23.1	16.9	10.6	14.8	18.0	54.8	68.5
10 days	23.1	16.9	10.4	14.8	18.4	105.8	116.9
12 days	23.0	16.8	10.5	14.9	18.5	126.7	167.8
14 days	23.1	16.9	10.5	14.9	18.5	158.3	Gelation

The thickness of silica AR coating synthesized by sol–gel method is dependent on the dip-coating withdrawal rate and sol viscosity. However, when the sol viscosity was low enough, no silica coating would be deposited on substrate during dip-coating process. Therefore, investigation on coating thickness is more direct than on sol viscosity for the purpose of monitoring particle growth. The coating thickness of varied H_2_O/TEOS molar ratio with a certain withdrawal rate at 100 mm min^−1^ was measured by ellipsometer. Table 2 shows the thickness of silica AR coating as a function of aging time and H_2_O/TEOS molar ratio. For the H_2_O/TEOS molar ratio value at 21.0, 15.0 and 9.0, the thickness of coatings also remained almost unchanged with aging time. The sol with H_2_O/TEOS molar ratio value of 3.0 and 2.1 could not be deposited within 2 days, thereafter extending the length of aging time; the thickness of the coating increased slightly and then almost remained stable with aging time. However, when H_2_O/TEOS molar ratio was 0.3 and 1.2, the sol could be deposited on substrate after 6 and 8 days aging, and then the corresponding coating thickness increased very fast, then gelation of the sol occurred.

**Table 2:** Change in film thickness with aging time at varied H_2_O/TEOS molar ratio

Aging time	The coating thickness (nm) of varied H_2_O/TEOS molar ratio						
	21	15	9	3	2.1	1.2	0.3
2 days	87.88	84.88	86.42	No film	No film	No film	No film
4 days	89.42	87.60	92.52	124.80	112.92	No film	No film
6 days	89.23	87.60	91.93	135.25	145.13	136.64	No film
8 days	88.65	86.05	90.55	130.94	156.78	159.48	88.50
10 days	86.10	87.40	91.34	125.41	152.54	177.59	126.77
12 days	88.65	87.60	92.91	125.61	155.08	207.76	194.47
14 days	90.58	88.37	93.70	129.51	159.32	253.45	Gelation
16 days	89.81	88.18	92.13	128.69	161.23	431.03	Gelation
18 days	89.76	88.17	92.35	128.54	162.11	Gelation	Gelation

### Particle morphologies of the gels

**Fig. 2:**
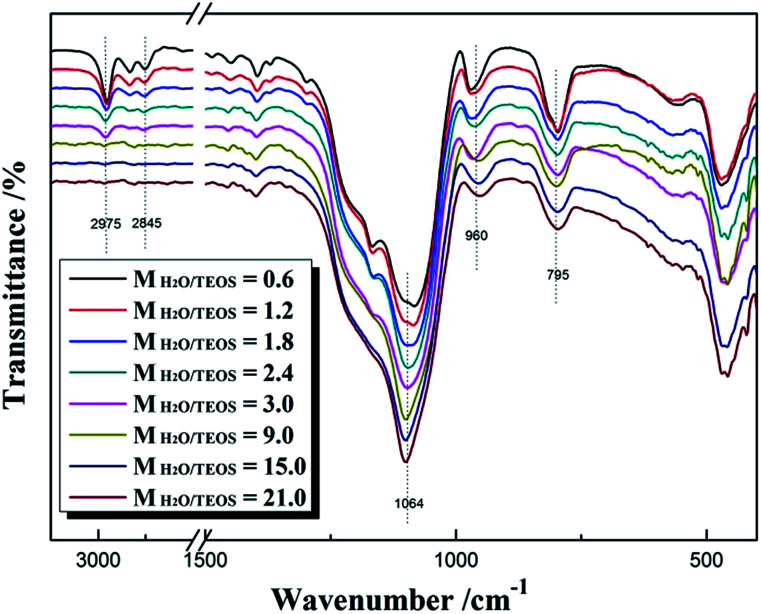
FT-IR spectra of different H_2_O/TEOS molar ratio.

Fig. 2 shows the FT-IR spectra of different *M*_H_2_O/TEOS_ coatings. As shown in Fig. 2, all silica samples have two absorption bands that are typical of silica from the sol–gel process at 1064 cm^−1^ and 795 cm^−1^. These bands are attributed to the Si–O–Si bond corresponding to bending and stretching vibrations, respectively. In Fig. 2, the absorption band at 960 cm^−1^ is attributed to the hydroxyl group of the silica particles. The absorption bands at 2975–2845 cm^−1^ is attributed to symmetric and asymmetric stretching vibration of C–H. As the water content increased, the absorption band at 2975–2845 cm^−1^ weakened gradually to disappeared, which could be concluded that the unhydrolyzed ethoxyl groups of coatings became less and less. It is deduced that as the H_2_O/TEOS molar ratio increased, the degree of TEOS hydrolysis enhanced; hence the silica particle was surrounded by more and more hydroxyl group.

**Fig. 3:**
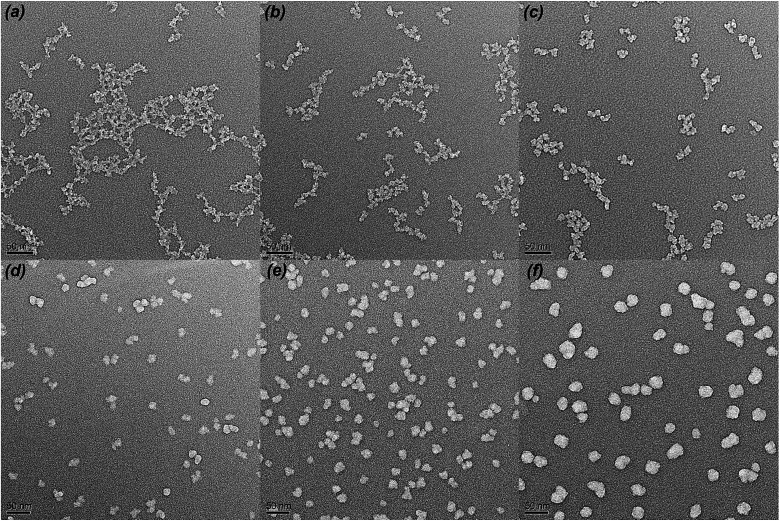
TEM images of H_2_O/TEOS molar ratio at 0.6 (a), 1.8 (b), 3.0 (c), 9.0 (d), 15.0 (e), 21.0 (f).

TEM was used to directly observe the shape and size of the silica particle at different H_2_O/TEOS molar ratio. As shown in Fig. 3, when the H_2_O/TEOS molar ratio was low, the silica particle was consisted of small size particle, and also showed different cross-linked degree. Especially, when the H_2_O/TEOS molar ratio was 0.6, the large silica particle showed greater cross-linked degree; however, the small silica particle showed smaller cross-linked degree. The different size silica particle simultaneously appeared in silica sol, which is in well agreement with the particle size and PDI results measured by DLS. As the H_2_O/TEOS molar ratio increased, the linear packing “net-work structure” disappeared and granular particle appeared gradually. When the H_2_O/TEOS molar ratio was larger than 9.0, the silica particle showed completely a granular particle structure and typical mono-dispersed system. And with the H_2_O/TEOS molar ratio increasing from 9.0 to 21.0, the diameter of granular silica particle increased. When the H_2_O/TEOS molar ratio were at 9.0, 15.0 and 21.0, the diameter of silica particle were 15.2 nm, 17.8 nm and 23.6 nm, respectively, these values were comparable with the results of DLS.

### Surface morphology of the coatings

**Fig. 4:**
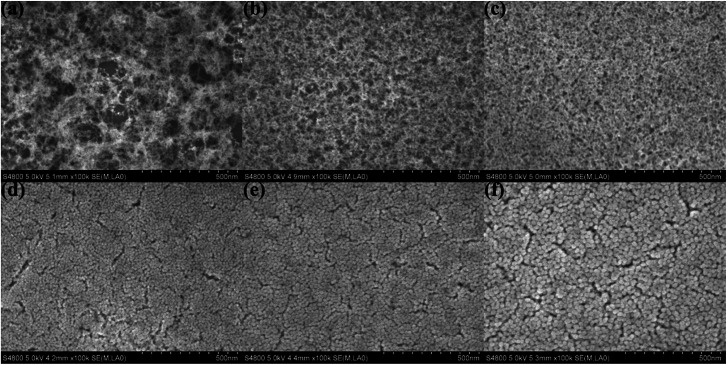
Surface SEM images of H_2_O/TEOS molar ratio being 0.6 (a), 1.8 (b), 3.0 (c), 9.0 (d), 15.0 (e), 21.0 (f).

The surface SEM was used to observe the stacking morphology of the silica particle, and the results are shown in Fig. 4. The coating of the H_2_O/TEOS molar ratio being 0.6 was deposited relatively thinly so as to obtain a more distinct image of the stacking silica structure. When the H_2_O/TEOS molar ratio was 0.6, the coatings showed “net-work structure”, and these “net-work structure” silica were consisted of some silica linear particle, which is consistent with the TEM image. As the H_2_O/TEOS molar ratio increased, the cross-linked structure disappeared gradually, granular particle appeared and turned bigger and bigger. When the H_2_O/TEOS molar ratio was 9.0, 15.0 and 21.0, all coatings showed almost uniform granular silica particle, whose diameter showed good agreement with the TEM results.

**Fig. 5:**
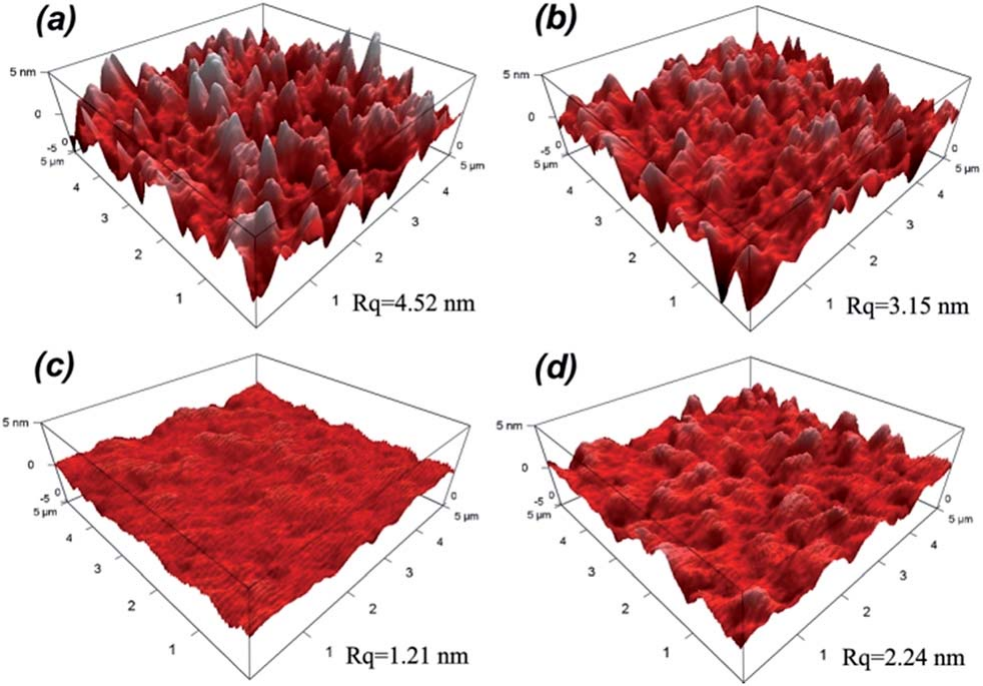
Surface morphologies of H_2_O/TEOS molar ratio being 0.6 (a), 1.8 (b), 9.0 (c), 21.0 (d).

The surface roughness has significant influence on optical performance of coatings. When the roughness dimension is much smaller than the light wavelength, the film/coating becomes increasingly transparent due to refractive index change between air and the coating, which effectively reduces the intensity of refraction at the air/film interface and increases the optical performance; hence the measured refractive index of coating is valid. In other words, it is necessary to control the roughness below 100 nm to effectively lower the intensity of Mie scattering.^35^ The surface morphologies of silica coatings with different H_2_O/TEOS molar ratio were analyzed by atomic force microscopy (AFM). As shown in Fig. 5, the H_2_O/TEOS molar ratio being 0.6, some sharp protuberance appeared at the surface of silica coating. As the H_2_O/TEOS molar ratio increased to 9.0, the sharp protuberance gradually disappeared and the surface of coating turned smooth. With the H_2_O/TEOS molar ratio increasing to 21.0, there appeared the blunt peak on surface of silica coating. This phenomenon could be interpreted by sol particle size and morphology of silica particle. When the H_2_O/TEOS molar ratio was low, that is 0.6; the coating was formed with linear packing net-work silica particle. Random stacking of linear packed net-work silica particle resulted in this sharp protuberance on the surface of coating. With the increase of H_2_O/TEOS molar ratio, net-work silica particle turned into granular silica particle. Especially at a higher H_2_O/TEOS molar ratio of 9.0, the diameter of the granular silica particle was 10.82 nm, its stacking resulted a quite smooth coating's surface. When the H_2_O/TEOS molar ratio was further increased to 21.0, bigger granular silica particles were formed, therefore, the corresponding coating's surface showed blunt peaks. When molar ratio of H_2_O/TEOS is 0.6, the root-mean-square (RMS) roughness (*R*_q_) of silica coating is 4.52. It was believed that the big aggregates with structure of linear net-work could spread out very well on fused silica substrate. As the molar ratio of H_2_O/TEOS increased to 1.8, 9.0, 21.0, the corresponding *R*_q_ were 3.15 nm, 1.21 nm, 2.24 nm, respectively. The surface morphologies of different H_2_O/TEOS molar ratio silica coatings are in very good agreement with particle shape.

### The mechanism of particle growth

**Fig. 6:**
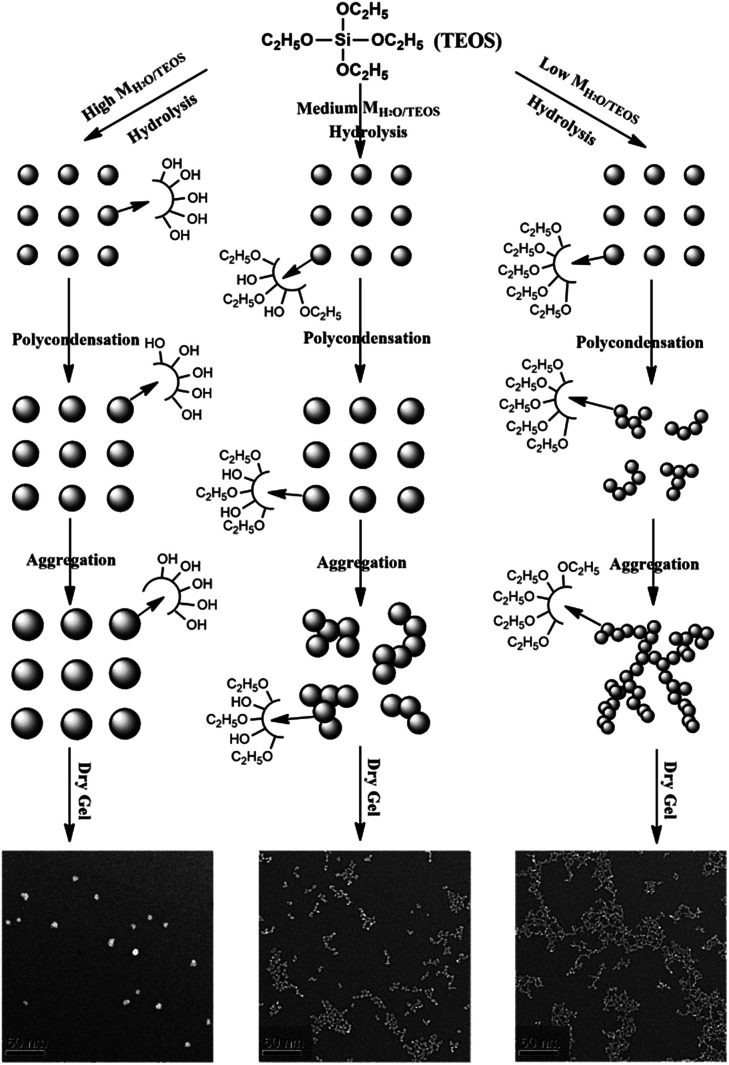
Schematic representation of the particle growth process of different H_2_O/TEOS molar ratio silica sols.

In the base-catalyzed condition, TEOS hydrolyzed and polymerized to form the silica sol or gel. According to Iler,^36^ polymerization occurs in three stages: (1) polymerization of monomer to form particles; (2) growth of particles; (3) linking of particles into chains, then networks that extend throughout the liquid medium, thickening it to a gel. However, as H_2_O/TEOS molar ratio varied, the hydrolysis of TEOS were different, hence obtained different morphology of structure. In this complex process, different H_2_O/TEOS molar ratio could be distinguished, which is shown in Fig. 6.

When H_2_O/TEOS molar ratio was low, through hydrolysis and primary polymerization, small-sized silica particle were formed in sol. However, because of low water content, these small-sized particle silica was surrounded by unhydrolyzed ethoxyl group. As the sol aging reaction continued, the some external ethoxyl groups of the silica particle were hydrolyzed to hydroxyl group and would most probably condense with hydroxyl group of another silica particle. The “net-work structure” silica particle was then formed and stopped until the silica sol get gel.

With the increase of H_2_O/TEOS molar ratio, after hydrolysis and primary polymerization, these silicon precursors trended to generate bigger-sized particle silica. The growth of particle stopped when silica particle were surrounded by enough non-substituted ethoxyl group. As the sol aging reaction continued, these bigger-sized silica particles reacted with each other to form some “bead-like structure” particle silica.

When the H_2_O/TEOS molar ratio was further increased to excess, more and more TEOS molecules were hydrolyzed into the tetra-hydroxyl-substituted silicon precursors. The polymerization maximized the number of Si–O–Si bonds and minimized the number of terminal hydroxyl groups through internal condensation to create the three-dimensional particle – “granular particle”. The growth stopped when the difference in the solubility between the smallest and the largest particle became negligible.^37^ The size distribution of the “granular particle” silica was monodispersed, which is confirmed by DLS experiments. The mechanism of particle growth showed good consistency with the TEM images of different H_2_O/TEOS molar ratio silica.

### Refractive indices of coatings

**Fig. 7:**
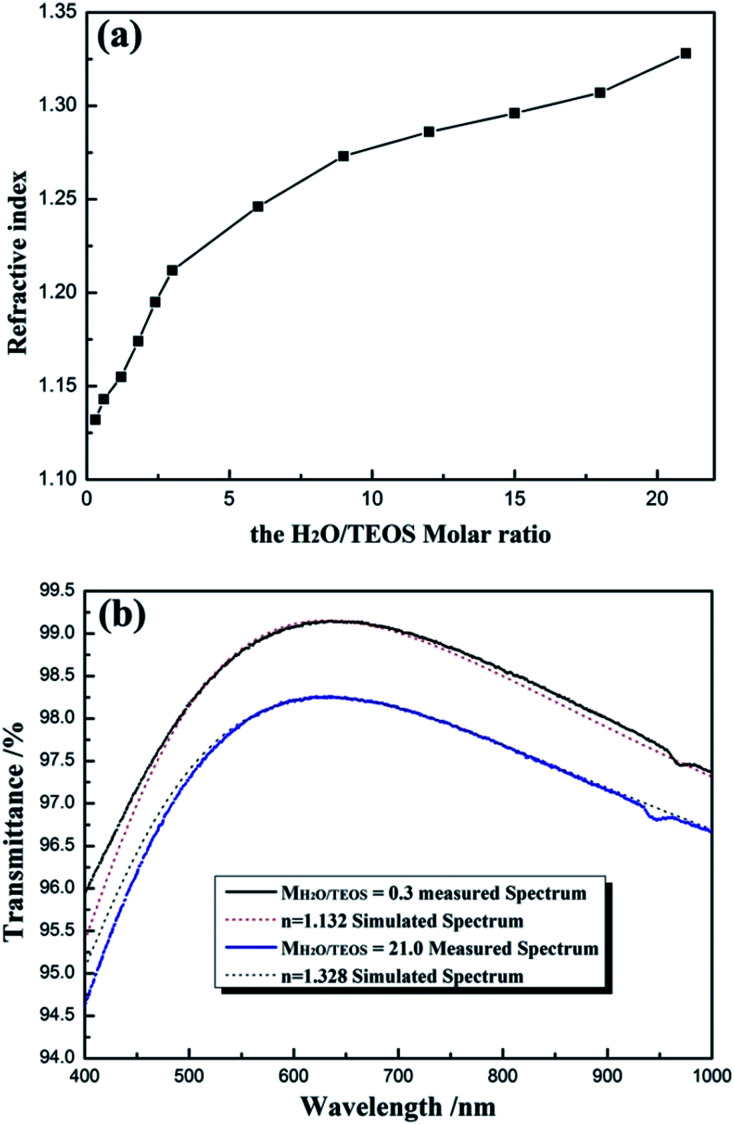
Refractive indices of AR coatings as a function of H_2_O/TEOS molar ratio (a), the measured transmittance spectrum of coatings with H_2_O/TEOS molar ratio being 0.3 and 21.0 and the simulated spectrum of coatings with *n* = 1.132 and *n* = 1.328 (b).

For obtaining AR coating with excellent transmittance, the refractive index of coating must match that of the substrate. For various substrates, it is significant to investigate the preparation of the coatings with continuous controllable refractive indices. Fig. 7(a) shows the refractive indices of coatings as a function of H_2_O/TEOS molar ratio. As shown in Fig. 7(a), as the H_2_O/TEOS molar ratio increased from 0.3 to 21.0, the refractive indices of coatings increased gradually from 1.132 to 1.328. While H_2_O/TEOS molar ratio increased from 0.3 to 3.0, the refractive indices of coatings increased rapidly, however, with the further increase of H_2_O/TEOS molar ratio up to 21.0, the corresponding refractive indices increased comparatively slowly. Once deposited as mere one-layer AR coating, such wide range of refractive indices could satisfy substrate with refractive indices ranging from 1.28 to 1.76 to obtain 100% transmittance. These coatings would also provide more option for multi-layers broadband antireflective films.

As all the measured coatings have been designed as quarter-wave coatings, *i.e.* with a thickness equivalent to one-quarter of the wavelength of the incident light, their transmittance can be used to evaluate the optical properties of the coatings according to the simplified Fresnel formula.^5^Fig. 7(b) shows the measured transmittance spectrum of coatings derived from the sol with *M*_H_2_O/TEOS_ being 0.3 and 21.0 and the simulated spectrum from software for the design of thin films (TFCalc, Version 3.5, Software Spectra Inc., USA) with *n* = 1.132 and *n* = 1.328, respectively, by setting the reference wavelength at ∼632 nm. It is found that the experimental and simulated curves show good agreement, which is evidence that the measured refractive indices of silica coatings were valid.

### Wettability property of AR coatings

**Fig. 8:**
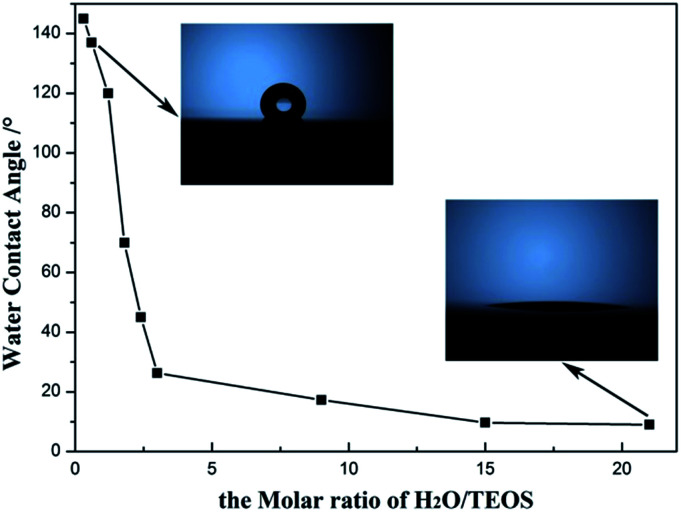
Static water contact angle values *versus* the H_2_O/TEOS molar ratio.

Hydrophobicity and hydrophilicity of AR coating, which can be monitored by the water contact angle, are important factor to value the environment response. The change of the water contact angle values of the AR coatings with H_2_O/TEOS molar ratio is shown in Fig. 8. As the H_2_O/TEOS molar ratio increased from 0.3 to 21.0, the static water contact angles of the AR coatings reduced from 145° to 6°. When the H_2_O/TEOS molar ratio was lower than 1.5, the static water contact angle of coatings was larger than 90°, which could be considered as hydrophobic. However, while the H_2_O/TEOS molar ratio exceeded 1.5, the static water contact angles of coatings were smaller than 90°, which implied some hydrophilic property. Especially, when the H_2_O/TEOS molar ratio was 21.0, the coating became very hydrophilic with a static water contact angle of 6°. Thus, by controlling the H_2_O/TEOS molar ratio, the coatings with hydrophobic or hydrophilic property can be prepared.

This change from hydrophobicity to hydrophilicity is attributed to the hydrolytic degree of TEOS. As the H_2_O/TEOS molar ratio increased, the degree of TEOS hydrolysis enhanced, hence the silica particle was surrounded by more and more hydroxyl group. Combined with the FT-IR spectra of different H_2_O/TEOS molar ratio, it is found that the change from hydrophobicity to hydrophilicity was caused by different degree of TEOS hydrolysis.

## Conclusions

By adjusting the H_2_O/TEOS molar ratio, silica coatings were prepared with controllable refractive indices and wettability properties. With the increase of H_2_O/TEOS molar ratio, the prepared silica particle possessed different morphologies, from “net-work structure” to “bead-like structure” and “granular structure” silica. When the H_2_O/TEOS molar ratio was 0.3, the refractive index was 1.132, as the H_2_O/TEOS molar ratio increased to 21.0, the refractive indices of silica coating gradually increased to 1.328. As the H_2_O/TEOS molar ratio increased from 0.3 to 21.0, the hydrolysis of TEOS increased, so the static water angle of silica coating decreased from 145° to 6°. These coatings with controllable refractive indices can satisfy a range of optical substrate and may be a good option for multi-layers broadband antireflective films and controllable wettability properties could be satisfactory to the different need of wettability behaviors.

## Conflicts of interest

There are no conflicts to declare.

## Supplementary Material
